# Synthesis, decom­position studies and crystal structure of a three-dimensional CuCN network structure with protonated *N*-methyl­ethano­lamine as the guest cation

**DOI:** 10.1107/S2053229620004477

**Published:** 2020-04-17

**Authors:** Christopher Koenigsmann, Leena N. Rachid, Christina M. Sheedy, Peter W. R. Corfield

**Affiliations:** a Department of Chemistry, Fordham University, 441 East Fordham Road, Bronx, NY 10458, USA

**Keywords:** copper cyanide, 3D network, thermogravimetric analysis, crystal structure, decomposition study, ethano­lamine, cuprophilic inter­action, spiral configuration

## Abstract

The com­pound poly[2-hy­droxy-*N*-methyl­ethan-1-aminium [μ_3_-cyanido-κ^3^ *C*:*C*:*N*-di-μ-cyanido-κ^4^ *C*:*N*-dicuprate(I)]], {(C_3_H_10_NO)[Cu_2_(CN)_3_]}_*n*_ or [meoenH]Cu_2_(CN)_3_, crystallizes in the tetra­gonal space group *P*4_3_. The structure consists of a three-dimensional (3D) anionic Cu^I^CN network with noncoordinated protonated *N*-methyl­ethano­lamine cations providing charge neutrality. Pairs of cuprophilic Cu atoms are bridged by the C atoms of μ_3_-cyanide ligands, which link these units into a 4_3_ spiral along the *c* axis. The spirals are linked together into a 3D anionic network by the two other cyanide groups. The cationic moieties are linked into their own 4_3_ spiral *via* N—H⋯O and O—H⋯O hydrogen bonds, and the cations inter­act with the 3D network *via* an unusual pair of N—H⋯N hydrogen bonds to one of the μ_2_-cyanide groups. Thermogravimetric analysis indicates an initial loss of the base cation and one cyanide as HCN at temperatures in the range 130–250 °C to form CuCN. We show how loss of a specific cyanide group from the 3D CuCN structure could form the linear CuCN structure. Further heating leaves a residue of elemental copper, isolated as the oxide.

## Abstract 2

The crystal structure is reported of a three-dimensional anionic Cu^I^CN network with noncoordinated protonated *N*-methyl­ethano­lamine cations providing charge neutrality. Pairs of cuprophilic Cu atoms are bridged by μ_3_-cyanide groups which link these units into 4_3_ spirals along the *c* axis, and these are linked together by other cyanide groups. On heating the com­pound to 280 °C, a CuCN residue is formed, while further heating leaves a residue of elemental copper, isolated as the oxide.

## Introduction   

Copper cyanide networks have been studied extensively in light of their inter­esting and unpredictable topologies, their photoluminescence, and the possible applications of their physical properties (see, for example: Grifasi *et al.*, 2016 ▸; Pike 2012 ▸; Dembo *et al.*, 2010 ▸; Tronic *et al.*, 2007 ▸). The Cu^I^CN–base networks in the literature either involve the base coordinated to Cu or the CuCN network carrying a net negative charge, requiring a cation in the network to provide neutrality. Our program of structural studies on mixed-valence copper cyanide com­plexes has sought to prepare neutral CuCN networks by incorporating divalent copper ions into Cu^I^ net­works, the Cu^II^ atoms being stabilized by coordination to one or more chelating ligands in the form of nitro­gen bases. The synthesis and structural analysis of the title com­pound, poly[2-hy­droxy-*N*-methyl­ethan-1-aminium [μ_3_-cyanido-κ^3^ *C*:*C*:*N*-di-μ-cyanido-κ^4^ *C*:*N*-dicuprate(I)]], (**I**), arose from an initial attempt to prepare such a neutral mixed-valence com­plex by partial reduction of Cu^2+^(aq) with the cyanide ion in the presence of *N*-methyl­ethano­lamine (meoen) as the stabilizing chelating ligand, a method which has previously produced crystalline products when chelating di­amines were used (see, for example: Corfield & Sabatino, 2017 ▸). After many unsuccessful attempts, mixtures containing crystalline com­pound (**I**) resulted, as described in the *Experimental* (§2) section. Although we have been unable so far to prepare a crystalline mixed-valence copper cyanide com­pound involving this base, we developed modified procedures to synthesize pure com­pound (**I**) in light of its inter­esting structural properties. Detailed thermogravimetric analyses of (**I**) were carried out in order to understand the structure further. Prom­pted by this work, our laboratory is now carrying out similar studies on a number of other CuCN networks based upon *N*-alkyl­ethano­lamines.
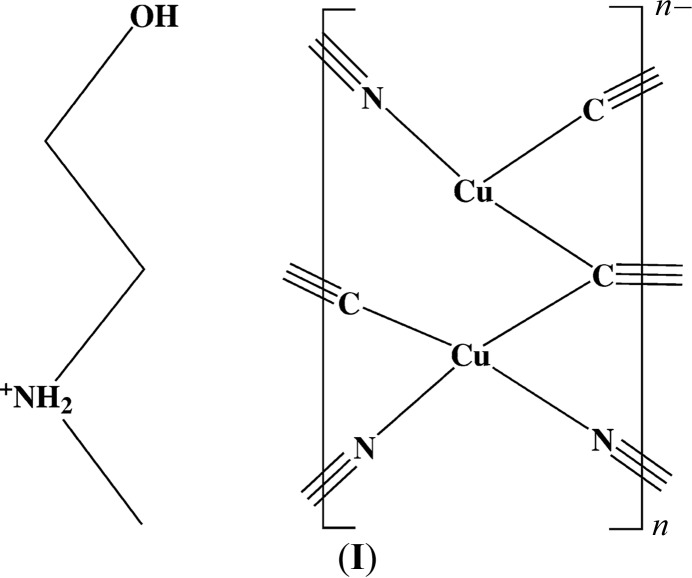


## Experimental   

Reagents were used as supplied by the manufacturers without further purification. IR spectra were obtained with a Thermo Scientific Nicolet iS50 FT–IR instrument, while thermal degradation studies were carried out under nitro­gen with a TA Instruments TGA-Q500 instrument. Scanning electron microscopy images were obtained with a Zeiss EVO MA-10 instrument equipped with an LaB6 filament. Images were collected with an accelerating voltage of 15 kV in variable pressure mode with a system vacuum of 40 Pa. SEM samples were prepared by drop-casting the powders dispersed in hexa­nes onto ultraflat p-doped Si wafers, which were dried under vacuum before analysis. Powder X-ray diffraction measurements were made with a Bruker D8 Advance Eco instrument. Elemental analyses were performed by Robert­son–Microlit Laboratories, Ledgewood, NJ, USA.

### Synthesis and crystallization   

**Figure 1:**
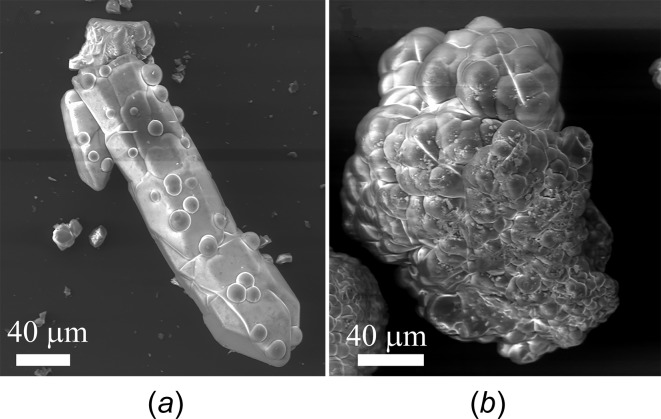
Electron microscope image showing (*a*) the crystalline and (*b*) the amorphous material in the initially synthesized heterogeneous green sample.

[meoenH]Cu_2_(CN)_3_ was initially synthesized by adding NaCN to solutions containing CuSO_4_ and *N*-methyl­ethano­l­amine, with the expectation of partial reduction of the Cu^II^ com­ponent to Cu^I^ and formation of our desired mixed-valent copper cyanide network. In general, preparations yielded heterogeneous green powders. The following preparation was the first to yield any crystalline material: CuSO_4_·5H_2_O (0.506 g, 2.03 mmol) was dissolved in water (10 ml) and stirred with a solution of meoen (0.779 g, 10.4 mmol) in water (5 ml), resulting in a deep-purple solution. A solution of NaCN (0.0968 g, 1.97 mmol) in water (10 ml) was added and the mixture heated to near boiling over a period of 90 min. Filtration after one week yielded a light-green solid material. Examination of the material under an optical microscope revealed a mixture of microcrystalline conglomerates and material that seemed noncrystalline. Further examination with a scanning electron microscope (SEM) confirmed the pre­sence of two morphologies: one that was facetted and appeared more crystalline (Fig. 1 ▸ *a*) and one that appeared amorphous (Fig. 1 ▸ *b*). It was evident that small spherical domains of the amorphous material were on the surface of the facetted single crystal and polycrystalline material (Fig. 1 ▸ *a*), giving the sample a uniformly green color.

**Figure 2:**
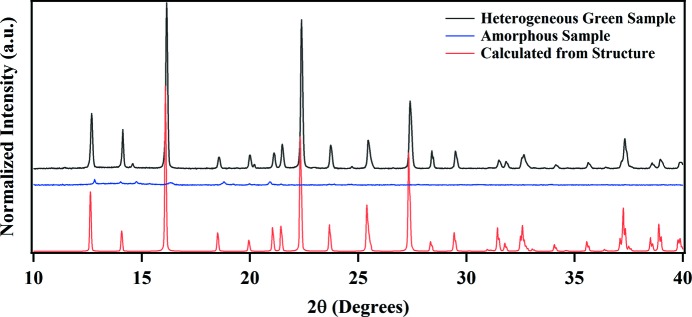
PXRD patterns for the heterogeneous green sample containing both crystalline and amorphous material (black), the sample containing the isolated green amorphous material (blue), together with the PXRD pattern expected from our single-crystal structure of [meoenH]Cu_2_(CN)_3_ (red), calculated from *GSAS II* (Toby *et al.*, 2013 ▸).

A small single crystal was selected after laborious examination of the sample. X-ray diffraction data were collected and data analysis resulted in a structure essentially the same as that reported in this article. The structure is made up of an anionic Cu^I^CN network, with noncoordinated protonated meoen mol­ecules providing charge neutrality; the mol­ecular formula is [meoenH]Cu_2_(CN)_3_. H atoms bonded to N and O atoms were visible in difference maps and could be refined, confirming that the meoen base was protonated and that each of the Cu atoms in the structure was monovalent. However, a small sample of the product gave an electron spin resonance (ESR) signal clearly indicating the presence of Cu^II^ in the sample. The majority of the remaining material was then used to obtain a powder X-ray diffraction (PXRD) pattern (Fig. 2 ▸). All peaks in the PXRD pattern matched the peaks expected from the single-crystal structure and there was little or no evidence for a second diffracting phase. It appeared that the ESR signal observed for the heterogeneous mixture was from the green amorphous com­ponent observed in the SEM images, and that this com­ponent did not give a PXRD pattern. In order to confirm this conclusion, syntheses along the original lines were repeated until a green noncrystalline product was obtained, shown by optical microscopy and SEM analysis to consist almost com­pletely of the previously identified amorphous morphology shown in Fig. 1 ▸(*b*). The PXRD pattern of this sample did indeed show mostly background scattering, and only one or two minor peaks (Fig. 2 ▸).

The synthetic procedure was modified to increase the likelihood of the formation of a pure sample of the crystalline com­pound by adding meoenH^+^ as the chloride salt to NaCN/CuCN mixtures instead of as the meoen base. A typical preparation such as the following produces a colorless crystalline product: the base meoen (0.751 g, 10.0 mmol) in water (30 ml) was titrated with 1 *M* HCl (11 ml) to a pH of 4.2; to a solution of NaCN (0.196 g, 4.0 mmol) in water (20 ml) was added CuCN(s) (0.224 g, 2.50 mmol) and further water (10 ml), and the mixture was stirred until all of the solid dissolved; the two solutions were combined and left to evaporate slowly. Colorless crystals of [meoenH]Cu_2_(CN)_3_ developed over a period of one or two weeks. Data crystals **3** and **4** were taken from such product samples and were shown to have exactly the same structure as found previously. These samples were ESR silent, as expected. Elemental analysis (%) on a colorless sample gave C 25.42, H 3.27, N 19.72, and Cu 45.14, agreeing well with the calculated values of 25.62, 3.58, 19.92, and 45.19%, respectively. Strong IR stretches were observed at 2083 and 2104 (C≡N), and 3110 and 3167 cm^−1^ (N—H). There was a sharp peak at 3529 cm^−1^ (hydrogen-bonded O—H) superimposed upon a broad peak in the O—H stretching region.

### Refinement   

Crystal data, data collection and structure refinement details for the title com­pound are summarized in Table 1 ▸. We collected data from crystals taken from four separate preparations of the com­pound, in order to confirm the identity of samples of both the green and colorless crystalline com­pounds described above. Each set of data was successfully refined in the space group *P*4_3_ with Flack parameters indistinguishable from zero. Refinement in the enanti­omeric space group *P*4_1_ gave significantly higher *R* factors, and Flack parameters of 1.0. Details of the four data sets and their refinements are presented in the supporting information. Reflection data from the four crystals were merged with the program *SORTAV* (Blessing, 1989 ▸), giving *R*_int_ = 0.0426, with the expectation that the use of averaged data from the four crystals would give an improved structure, as well as clarifying the H-atom positions on the disordered hy­droxy group. [Data for each crystal were first averaged with *SCALEPACK* (Otwinowski & Minor, 1997 ▸) in the *KappaCCD server software* (Nonius, 1997 ▸).] The crystal size given in Table 1 ▸ is given for crystal **4**. Crystals **1** and **2** were of similar size, but crystal **3** was much larger than the others; data from this crystal were used to help improve signal-to-noise ratios for the weaker reflections. In all, 14 469 intensities processed from the four crystals were merged to give the 3908 data used in this study. *SORTAV* rejected 407 reflections from the merge based upon statistical tests, including 371 stronger reflections from crystal **3** which were rejected because they had significantly less intensity than reflections from the other crystals, presumably due to detector saturation for the strong reflections from the larger crystal **3**.

CN occupancies for the C and N atoms of the cyanide groups were refined at a late stage. None of the C/N occupancy factors differed significantly from 100/0%, so that we have refined all of the cyanide groups as ordered. In the case of the μ_3_-bridging cyanide C3≡N3, this ordering was expected, as the C and not the N atom is usually preferentially bound to the pair of close Cu atoms in such cases. The clear choice of orientation taken by the two other cyanide groups is possibly due to inter­actions with the cations, as discussed below.

Methyl­ene H atoms in the *N*-methyl­ethano­lamine moiety were constrained to positions calculated assuming a tetra­hedral geometry at the C atom and a C—H distance of 0.97 Å. H atoms on the methyl group were constrained similarly, with C—H distances of 0.96 Å and the methyl group was allowed to rotate. We have modeled a 50:50 disordered hy­droxy group. The disordered hy­droxy H atoms and the two H atoms on the charged N atom were allowed to refine individually, with restraints on the N/O—H distances, and displacement parameters were constrained to be equal for the two pairs.

## Results and discussion   

### Description of the structure   

**Figure 3:**
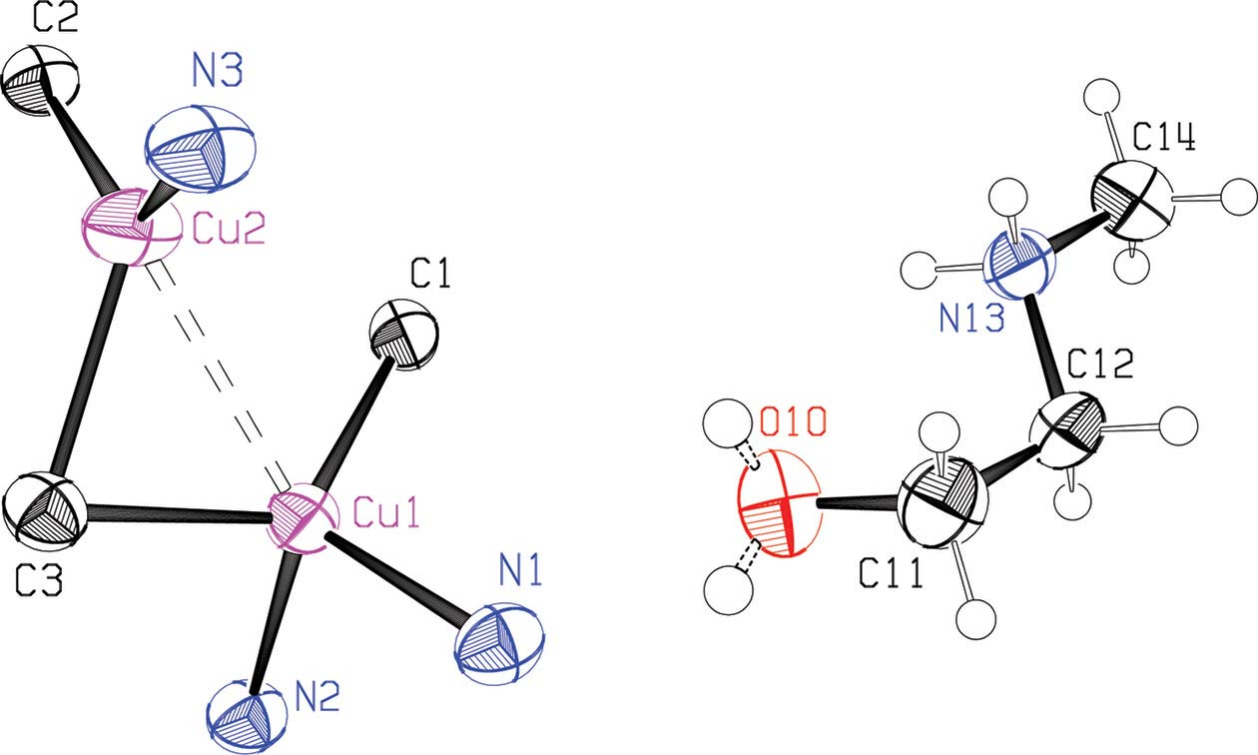
The asymmetric unit of the title com­pound, showing displacement ellipsoids for heavier atoms at the 70% probability level and H atoms at an arbitrary scale. Both of the disordered hy­droxy O atoms are shown. The dashed line indicates the cuprophilic inter­action. In this figure, as well as in Figs. 4 ▸ and 5 ▸, the color code is: Cu magenta; O red; N blue; C black.

Displacement ellipsoids for the asymmetric unit of the structure are shown in Fig. 3 ▸. The structure is com­posed of a three-dimensional (3D) anionic Cu^I^ network, with the mol­ecular formula [Cu_2_(CN)_3_]^−^. Charge neutrality is maintained by protonated *N*-methyl­ethano­lamine mol­ecules, [meoenH]^+^, situated in the network cavities. The CuCN network is built up from dimeric Cu_2_(CN)_6_ units (see Scheme), with the two Cu atoms in each unit held together in a cuprophilic inter­action by one μ_3_-bridging CN group (Fig. 3 ▸). The Cu⋯Cu distance is 2.5822 (5) Å and the two Cu—C distances to the μ_3_-C atom are 2.007 (2) and 2.138 (2) Å. The Cu⋯Cu distance is usually found to be shortest when bridging distances are more symmetrical (Stocker *et al.*, 1999 ▸; Corfield *et al.*, 2016 ▸). In this case, the Cu—C distances differ significantly and the Cu⋯Cu distance is inter­mediate, perhaps also because there is only one μ_3_-bridging cyanide instead of the two that are often found. Of the two Cu atoms, Cu1 is bound to four cyanide groups in a distorted tetra­hedral arrangement, with bond angles ranging from 97.87 (9)° for N2—Cu1—C3 to 124.80 (10)° for C1—Cu1—C3. The Cu2 atom is trigonally bound to three cyanide groups, with bond angles of 104.47 (10)° for C3—Cu2—N3, 121.26 (10)° for C2—Cu2—C3, and 127.14 (10)° for C2—Cu2—N3. The sum of the bond angles around Cu2 is 352.87 (6)°, which implies a significant distortion from planarity for this atom. The Cu—C/N distances vary from 1.910 (2) to 2.138 (2) Å, with a mean of 2.02 (3) Å. Each of the three CN groups bridges Cu atoms, with a mean C—N distance of 1.147 (2) Å and angles within 12° of linearity for the two μ_2_-CN groups. There are no terminal CN groups.

**Figure 4:**
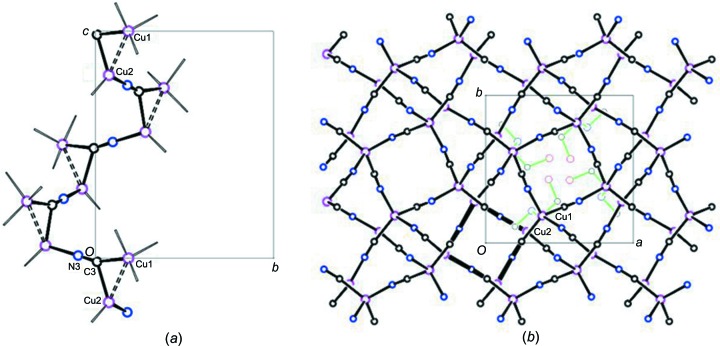
(*a*) Projection along the *a* axis of the spiral chain of Cu_2_(CN)_5_ and C3≡N3 units related by the 4_3_ axis at *x* = 0, *y* = 0. Generic atom labels without symmetry codes have been used. (*b*) Packing diagram showing a projection along the *c* axis. The projection of the spiral chain shown in Fig. 4 ▸(*a*) is highlighted in bold. Only the cations within the unit cell are shown, with bonds drawn in green.

The μ_3_-C3≡N3 group of the asymmetric unit bridges to a Cu2 atom related by the 4_3_ crystallographic axis at *x* = 0, *y* = 0, so as to link the dimers into a spiral chain (Fig. 4 ▸ *a*). Fig. 4 ▸(*b*) shows a projection of the CuCN network down the *c* axis, with this spiral structure bolded. Cyanide groups C1≡N1 also link Cu1 atoms into a spiral related by the 4_3_ axis at *x* = 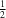, *y* = 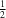, seen as a square structure inside the unit-cell outline in Fig. 4 ▸(*b*). The C2≡N2 cyanide groups link these two sets of spirals into the 3D network.

**Figure 5:**
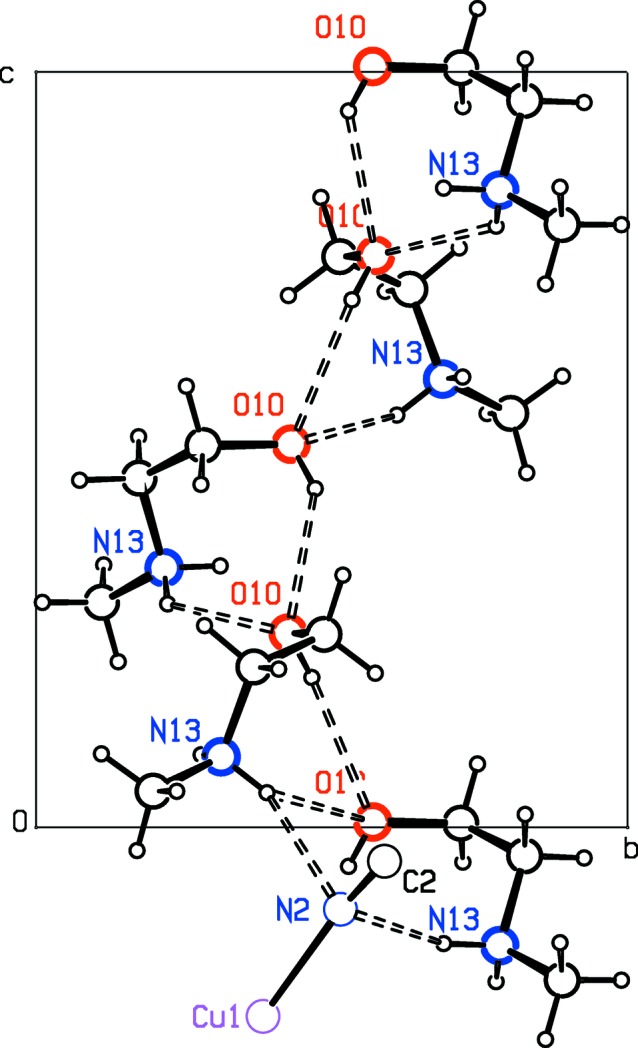
Projection along the *a* axis of the spiral chain of hydrogen-bonded meoenH^+^ ions. Only one of the disordered hy­droxy O atoms is shown.

The hy­droxy O atom in the guest cation is in a *gauche* configuration with respect to the extended conformation of the rest of the mol­ecule. These meoenH^+^ cations are linked *via* N—H⋯O and O—H⋯O hydrogen bonds into their own spiral structure around the 4_3_ axis at *x* = 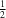, *y* = 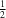 (Table 2 and Fig. 5 ▸). Formation of the O—H⋯O hydrogen bonds requires that the disordered –OH groups assume a *cis* conformation with respect to the neighboring CH_2_ group. The disordered model for the hy­droxy H atoms mean that O—H⋯O hydrogen-bonded chains can point either up or down the spiral chains. In addition, there are hydrogen-bonding N—H⋯N inter­actions between the protonated amine groups and the N atom of bridging cyanide C2≡N2 of the CuCN anionic 3D structure (Fig. 5 ▸), which doubtless favors the orientation found for this cyanide group in the structure. The N⋯N distances are 3.089 (3) and 3.093 (3) Å, and the N—H⋯N hydrogen-bond angles are 144 (3) and 152 (3)°. The N⋯N inter­actions are almost at right angles to the Cu—C≡N—Cu chain, with N13⋯N2—Cu1 and N13⋯N2—C2 angles of 97.48 (9) and 92.0 (2)° for the H13*A* hydrogen bond, and 94.54 (9) and 88.4 (2)° for the H13*B* hydrogen bond.

**Figure 6:**
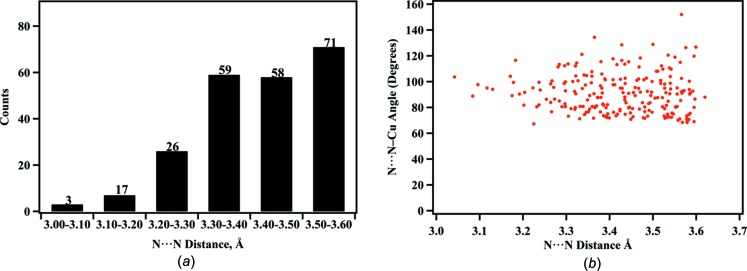
Histograms of N—H⋯Cu—C≡N—Cu contacts (*a*) and angles (*b*).

Intrigued by these unusual hydrogen-bond inter­actions, we explored occurrences of N—H and Cu—C≡N—Cu inter­actions in the Cambridge Structural Database (CSD, Version 2.0.3; Groom *et al.*, 2016 ▸). A search for such N⋯N contacts less than the sum of the van der Waals radii + 0.5 Å yielded 225 cases of such contacts in 109 entries, with 27 of the contacts involving N^+^—H hydrogen bonds, and the rest N—H inter­actions. A histogram of the N⋯N contacts found (Fig. 6 ▸ *a*) indicates only three structures with distances as close as seen in the present structure, indicating that this inter­action is rare, though not unique. While all of the N⋯N—Cu angles cluster broadly around 90°, Fig. 6 ▸(*b*) shows that angles close to 90° are found especially for shorter N⋯N distances. The shortest such N⋯N distance is found in the structure of [NH_3_(CH_2_)_5_NH_3_]_3_Cu_8_(CN)_14_·3H_2_O (Pretsch & Hartl, 2004 ▸). Here, one of the Cu—C≡N—Cu chains has two such contacts to the cyanide N atom in the chain, at 158° to one another. The N⋯N distances are 3.04 and 3.25 Å, and the N—H⋯N hydrogen-bond angles are 162 and 158°. One of the other examples of shorter N⋯N distances that we had not previously noted occurs in our own structure of [(C_2_H_5_)_2_NH(CH_2_)_2_OH]Cu_2_(CN)_3_ (Corfield *et al.*, 2016 ▸), where the N⋯N distance is 3.13 Å and the N—H⋯N angle is 145°.

### Thermal decom­position studies   

**Figure 7:**
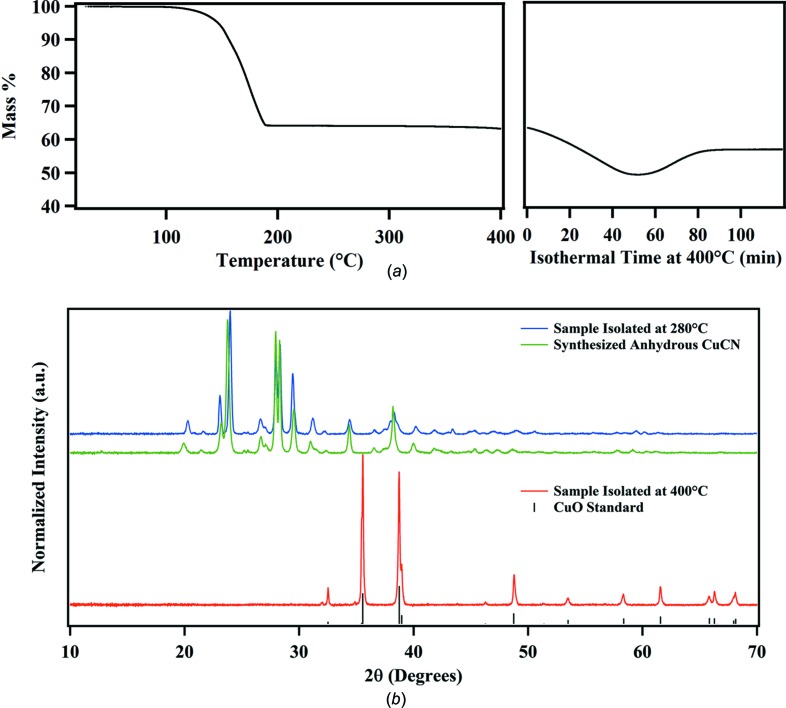
(*a*) Thermogravimetric analysis data for [meoenH]Cu_2_(CN)_3_. The temperature was ramped at 4 °C min^−1^ to 400 °C (left), producing the first mass loss of 36%. The temperature was held at 400 °C for 120 min to produce the second mass loss of 52% of the initial mass in an isothermal step (right). (*b*) PXRD data collected after TGA of samples heated to 280 and 400 °C. The PXRD pattern for our synthesized CuCN and a standard pattern for CuO (COD 90144580) are also shown.

Fig. 7 ▸(*a*) shows the percent mass loss when a sample of the title com­pound was heated at 4 °C min^−1^ to 400 °C and maintained at that temperature for 2 h. An initial loss of 36% is com­plete by 280 °C and corresponds to a mass loss of 101 u. This mass closely approximates the mass of the meoen moiety plus HCN (75.1 + 27.0 = 102 u) which would leave behind material with the com­position CuCN. The continued weight loss to 52% represents the loss of cyanide, leaving mostly elemental copper behind. The reproducible weight gain at the end of the experiment was puzzling. The black material remaining gave a PXRD pattern that matched that for copper(II) oxide (Fig. 7 ▸ *b*), and showed %N < 0.1% upon elemental analysis. We conclude that there must be some source of oxygen in the apparatus, either from a leak or from the nitro­gen cylinder. In a separate thermogravimetric analysis (TGA) experiment with 20 mg of sample, heating was stopped at 280 °C, and the cream-colored solid remaining was examined to verify its com­position. The IR spectrum of this solid showed CN stretches at 2122 and 2166 cm^−1^, identical to the CN stretching frequencies found in an IR sample of a freshly prepared CuCN sample. Elemental analysis (%) of the 280 °C residue was reported at C 13.52, H 0.16, and N 15.41, com­pared with the values of C 13.41, H 0, and N 15.64 expected for CuCN. The residue gave a PXRD pattern very similar to that for a sample of CuCN (Fig. 7 ▸ *b*). The 280 °C residue is clearly CuCN.

We propose a mechanism by which the 3D CuCN network structure shown in Fig. 4 ▸ could transform to the linear Cu—C≡N—Cu chains of CuCN on heating to 280 °C with loss of a cyanide and the guest base cation. We note that the spiral CuCN chains at *x* = 0, *y* = 0, shown in Fig. 4 ▸(*a*). involve a continuous Cu2—C3≡N3—Cu2—C3≡N3—Cu2 sequence, and that the spirals shown in Fig. 4 ▸(*b*) at *x* = 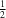, *y* = 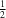 involve a continuous Cu1—C1≡N1—Cu1—C1≡N1—Cu1 sequence. These spirals are connected by the bridging cyanide C2≡N2 groups. The release of cyanide C2≡N2 from the structure, together with the breakage of the C3—Cu1 bond and the release of the Cu atoms from their cuprophilic inter­action can leave the two separate spirals free to reorganize into the linear structure found in the room temperature ortho­rhom­bic CuCN crystalline phase which was obtained.

We have also explored further the com­pound(s) lost during the initial heating phase in the TGA experiment. Samples of the title com­pound were heated in a glass sublimation apparatus to ∼200 °C, and the liquid sublimate on the water-cooled cold finger was collected. Carrying out the sublimation in air gave a com­plex mixture, as indicated by the IR and NMR spectra, as well as by GC/MS results. When the experiment was repeated under an argon atmosphere, the GC/MS results indicated a mixture of three species. The predominant com­pound (80%) had a fragmentation pattern that closely matched that expected for meoen, but it was not possible to identify the two minority com­pounds. The IR spectrum of the mixture included peaks expected for meoen, together with a strong peak at 1665 cm^−1^, which must be due to one of the minority species formed, perhaps by thermal decom­position of the hot base.

## Supplementary Material

Crystal structure: contains datablock(s) I, global. DOI: 10.1107/S2053229620004477/op3005sup1.cif

Structure factors: contains datablock(s) I. DOI: 10.1107/S2053229620004477/op3005Isup2.hkl

Supplemental data on the four individual crystals. DOI: 10.1107/S2053229620004477/op3005sup3.txt

CCDC reference: 1993944

## Figures and Tables

**Table 1:** Experimental details

Crystal data	
Chemical formula	(C_3_H_10_NO)[Cu_2_(CN)_3_]
*M*_r_	281.26
Crystal system, space group	Tetragonal, *P*4_3_
Temperature (K)	303
*a*, *c* (Å)	8.8994 (5), 11.3750 (11)
*V* (Å^3^)	900.89 (13)
*Z*	4
Radiation type	Mo *K*α
μ (mm^−1^)	4.68
Crystal size (mm)	0.30 × 0.07 × 0.07

Data collection	
Diffractometer	Enraf–Nonius KappaCCD
Absorption correction	Part of the refinement model (Δ*F*) (Otwinowski & Minor, 1997 ▸)
*T*_min_, *T*_max_	0.56, 0.77
No. of measured, independent and observed [*I* > 2σ(*I*)] reflections	3962, 3962, 3685
*R*_int_	0.043
(sin θ/λ)_max_ (Å^−1^)	0.806

Refinement	
*R*[*F* ^2^ > 2σ(*F* ^2^)], *wR*(*F* ^2^), *S*	0.025, 0.048, 1.08
No. of reflections	3962
No. of parameters	134
No. of restraints	8
H-atom treatment	H atoms treated by a mixture of independent and constrained refinement
Δρ_max_, Δρ_min_ (e Å^−3^)	0.49, −0.41
Absolute structure	Flack *x* determined using 1606 quotients [(*I* ^+^) − (*I* ^−^)]/[(*I* ^+^) + (*I* ^−^)] (Parsons *et al.*, 2013 ▸)
Absolute structure parameter	0.008 (8)

Com­puter programs: *KappaCCD Server Software* (Nonius, 1997 ▸), *DENZO* and *SCALEPACK* (Otwinowski & Minor, 1997 ▸), *SHELXT* (Sheldrick, 2015*a* ▸), *SHELXL2017* (Sheldrick, 2015*b* ▸), *ORTEPIII* (Burnett & Johnson, 1996 ▸), *ORTEP-3* (Farrugia, 2012 ▸) and *publCIF* (Westrip, 2010 ▸).
